# The strategies for the supplementation of vitamins and trace minerals in pig production: surveying major producers in China

**DOI:** 10.5713/ajas.20.0521

**Published:** 2020-11-03

**Authors:** Pan Yang, Hua Kai Wang, Long Xian Li, Yong Xi Ma

**Affiliations:** 1 State Key Laboratory of Animal Nutrition, College of Animal Science and Technology, China Agricultural University, Beijing 100193, China; 2 Ministry of Agriculture and Rural Affairs Feed Industry Centre, Beijing 100193, China

**Keywords:** China, Pig Industry, Supplementation Level, Trace Mineral, Vitamin

## Abstract

**Objective:**

Adequate vitamin and trace mineral intake for pigs are important to achieve satisfactory growth performance. There are no data available on the vitamin and trace mineral intake across pig producers in China. The purpose of this study was to investigate and describe the amount of vitamin and trace minerals used in Chinese pig diets.

**Methods:**

A 1-year survey of supplemented vitamin and trace minerals in pig diets was organized in China. A total of 69 producers were invited for the survey, which represents approximately 90% of the pig herd in China. Data were compiled by bodyweight stages to determine descriptive statistics. Nutrients were evaluated for vitamin A, vitamin D, vitamin E, vitamin K, thiamine, riboflavin, vitamin B_6_, vitamin B_12_, pantothenic acid, niacin, folic acid, biotin, choline, copper, iron, manganese, zinc, selenium, and iodine. Data were statistically analyzed by functions in Excel.

**Results:**

The results indicated variation for supplemented vitamin (vitamin A, vitamin D, vitamin E, vitamin K, vitamin B_12_, pantothenic acid, niacin, and choline) and trace minerals (copper, manganese, zinc, and iodine) in pig diets, but most vitamins and trace minerals were included at concentrations far above the total dietary requirement estimates reported by the National Research Council and the China’s Feeding Standard of Swine.

**Conclusion:**

The levels of vitamin and trace mineral used in China’s pig industry vary widely. Adding a high concentration for vitamin and trace mineral appears to be common practice in pig diets. This investigation provides a reference for supplementation rates of the vitamins and trace minerals in the China’s pig industry.

## INTRODUCTION

Although vitamins and minerals constitute a relatively small percentage of the diet, they are vital to health, well-being, and performance, each of them plays well defined metabolic roles, the essentials of which varies depending on the physiological stage of animals [1–5]. Further, vitamins and trace minerals are the foundation of balanced pig nutrition: Proper supplementation of micronutrients in the diet is important for maintaining the health and growth performance of pigs, and for reducing unnecessary costs [5,6]. The National Research Council (NRC) estimates the recommended intake of dietary vitamins and trace minerals [6,7]. In the past few decades, many studies have been conducted to determine the vitamin requirements of pigs and these have been used by the NRC, but there has been relatively little change in the requirement estimates over that time frame [6,8]. The Feeding Standard of Swine also recommends the amounts of vitamins and trace minerals in the diet of China’s pig producers [9]. In practice, most commercial diets are formulated with vitamin and trace mineral levels well above those recommended by the NRC and Feeding Standard of Swine, which take account of the potential variation and bioavailability of vitamins and trace minerals [4,10], fluctuations in the daily feed intake of animals [7,11], and degradation of vitamins due to transportation, storage, and processing [12,13]. In addition, two feeding guides (NRC and Feeding Standard of Swine) may not reflect greatly improved genetic selection and changes in management procedures of modern pig operations.

China is the world’s largest pork producer and consumer. Farmers and companies in China produce more than 50 million metric tons of pork every year (National Bureau of Statistics of China). The high productivity of intensive farming in China requires the optimal levels of growth and reproduction performances. Vitamins and trace minerals may need to be adjusted to meet the increased strength of production, but much of the limited information available on this topic is outdated. The objective of this study was to collect information regarding added the amount of vitamin and trace mineral supplementation in pig diets across China’s pig industry. We want to provide the information on a complete feed basis and include hand-added ingredients that have been used in formulations, to enable us to target more precise feeding during different phases of pig production.

## MATERIALS AND METHODS

The procedures for this survey were approved by the Ministry of Agriculture and Rural Affairs Feed Industry Centre Committee (Beijing, China). Approval from the Animal Care and Use Committee was not obtained for this experiment because no animals were used. The survey information was collected in the digital spreadsheet (Excel; Microsoft 365, Redmond, WA, USA).

### Procedure

**Figure 1:**
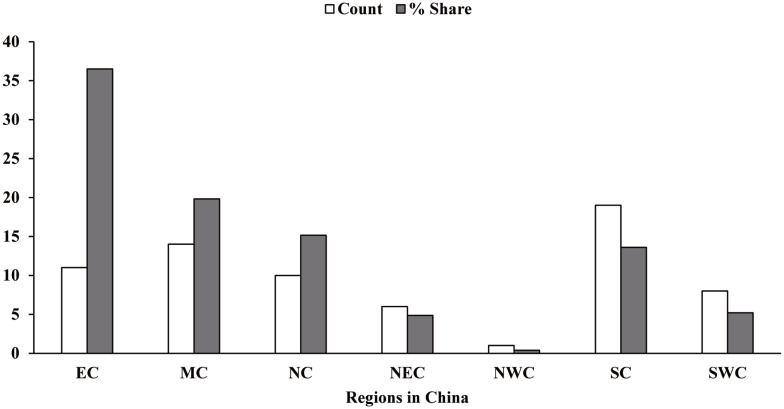
Distribution and market share of producers in China; EC, East China; MC, Middle China; NC, North China; NEC, Northeast China; NWC, Northwest China; SC, South China; SWC, Southwest China.

The subjects of the investigation were pig producers within China. Most of the large producers in China were involved in this investigation, and the survey was completed from the period of January to December 2019. A total of 69 producers agreed to be involved with the investigation; the distribution and market share of these producers involved in the survey are presented in Figure 1. This survey sampled information from approximately 90% of China’s pig herd. This pig production systems included most parts of mainland China. Questionnaires were distributed *via* E-mail and Wechat (Tencent Holdings Ltd., Shenzhen, China), which consisted of a survey covering major aspects of approximate weight breaks for feeding phases, the premix specifications (manufacturers of vitamins usually provide concentrated supplements known as premixes), inclusion rates of added vitamins and trace minerals, and response evaluation and outcomes.

### Feeding phases division, vitamins, and trace minerals

Different producers have different feeding stages and approximate dietary weight changes. However, the results were relatively consistent across a wide range of weights among all producers participating in this survey. The NRC is the most famous publication in pig nutrition and established the recommended requirement of vitamins and trace minerals for pigs, but for different body weight stages, the vitamins and trace minerals required for pigs according to the NRC does not change much. In addition, we considered the specific status in China’s animal husbandry, and the official recommendation of feeding standard in China [9] and application in production. The feeding phases were divided into five stages of pig production including creep, nursery, growing, finishing, and breeding phrases. The creep diets consisted of weanling to 8 kg body weight; the nursery diets consisted of early-phase (8 to 15 kg) and later-phase (15 to 25 kg) pigs; the growing diets consisted of 25 to 60 kg body weight animals; the finishing diets consisted of early-finishing (60 to 90 kg) and late-finishing (90 kg to market) pigs; and breeding herd diets consisted of gilt development (25 kg to breeding), gestation, lactation, and boar. Within each feeding phase, the vitamins and trace minerals were evaluated vitamin A, vitamin D, vitamin E, vitamin K, thiamine, riboflavin, vitamin B_6_, vitamin B_12_, pantothenic acid, niacin, folic acid, biotin, choline, copper, iron, manganese, zinc, selenium, and iodine.

### Statistical and analysis

Basal information about producers was visualized using Microsoft Office 365 Excel (Microsoft Corporation, Redmond, WA, USA) and Adobe Illustrator 2020 (Adobe Inc., San Jose, CA, USA). Data were compiled, pooled, and reviewed by all members prior to publication. All values were determined by descriptive statistics using functions in the pivot tables in Excel (Microsoft 365, USA) and included average (AVERAGE), standard deviation (STDEV.S), median (MEDIAN), minimum (MIN), maximum (MAX), and the 25th and 75th percentiles (QUARTILE.EXC). The NRC provides estimates of the amounts of vitamins and trace minerals for various classes of pigs under average conditions. However, factors such as genetic variation, environment, availability of nutrients in feedstuffs, and other stressors may increase the concentration of some nutrients required for optimal reproduction performance. The vitamins and trace mineral estimation in China’s Feeding Standard of Swine may be more specific for the pig industry in China. Thus, the average supplementation rate of each nutrient was calculated as two ratios to the suggested requirement as provided by the NRC [7] and China’s Feeding Standard of Swine [9], respectively. This comparison was used to explore the status of vitamin and trace mineral utilization in China. We also calculated minimum and maximum inclusion rates as a proportion to these two requirement guides (data not shown). The NRC and China’s Feeding Standard of Swine all provide total dietary vitamin and trace mineral requirements, while the results were presented that vitamins and trace minerals provided the following per kilogram of diet for each physiological stage of pigs.

## RESULTS

### Creep stage

The supplementation rates of vitamins and minerals in the creep diets are presented in Table 1. The data was provided by 155 pig farms belonging to 37 producers. The average fat-soluble vitamin supplementation rate was 1.84 to 16.1 times higher than the estimated requirements published by the NRC and China’s Feeding Standard. Vitamin D was supplemented at 16.1 times that of the NRC requirement estimate and China’s Feeding Standard, and the supplementation of vitamin D had relatively high variation (standard deviation [SD] = 1,726.678). The highest amount of variation (SD = 3,848.980) occurred in vitamin A supplementation among producers. The average amount of supplemented vitamins E was close to their requirements (1.84 times), and the average supplemented vitamins A and K were 4.67 and 8.95 times that of NRC or China’s Feeding Standard. Water-soluble vitamins were supplemented from 0.47 to 9.25 times for their NRC requirement estimates, and 0.75 to 9.25 times for the Feeding Standard. Vitamin B_6_, niacin, and choline was supplemented on average below the estimation of their requirements. Trace minerals were supplemented from 1.35 to 17.6 times over the NRC requirement estimate and China’s Feeding Standard. Selenium was supplemented just over the requirement estimates (1.35 times). Supplemented iron and zinc were close to the NRC recommendation (2.58 and 4.25 times, respectively) and China’s Feeding Standard (2.45 and 3.86 times, respectively). Copper, manganese, and iodine were supplemented well above the requirements at 13.7, 17.6, and 13.7 times over the recommendations of the NRC and China’s Feeding Standard, respectively. The minimum vitamin and trace mineral contents were lower than NRC and China’s Feeding Standard estimates, the average of their maximum supplementation was 48.7 and 48.9 times that of NRC and China’s Feeding Standard, respectively.

### Nursery stage

The early-nursery pig diet supplementation rates of vitamins and trace minerals are provided in Table 2. The 57 companies supplied feed to 327 pig farms, which were surveyed in this study. The average fat-soluble vitamin supplementation rate was 2.12 to 12.9 times higher than the NRC requirement estimates, and 3.08 to 14.1 times higher than China’s Feeding Standard requirement. Vitamin D supplementation was 12.9 times higher than the NRC requirement estimate and 14.1 times higher than China’s Feeding Standard. A high amount of variation (SD = 3,124.882) was observed in vitamin A supplementation across producers. The supplementation of vitamins A, E, and K on average was close to the requirements, at 4.63, 2.12, and 6.91 times higher than the NRC; at 5.66, 3.08, and 6.91 times higher than China’s Feeding Standard, respectively. Water-soluble vitamin supplementation was from 0.42 to 7.95 times higher than the NRC requirement estimate, and 0.69 to 7.95 times higher than China’s Feeding Standard. The average vitamin B_6_ and niacin supplementation were below the NRC requirement by 0.42 and 0.88 times, respectively, but at their requirement estimate in China’s Feeding Standard by 1.94 and 1.76 times, respectively. Choline supplementation was 0.69 times lower than the NRC and China’s Feeding Standard. Trace minerals supplementation was from 1.23 to 15.7 times higher than the NRC requirement estimate and China’s Feeding Standard. Selenium supplementation met the requirements. Iron, zinc, and iodine were added at levels close their requirements (2.44, 5.34, and 8.91 times higher than the NRC requirement, respectively, and 2.32, 4.86, and 8.91 higher than China’s Feeding Standard, respectively), and copper and manganese were supplemented well above their requirement estimate, at 12.8 and 15.7 times that of the two feeding recommendations, respectively. The minimum vitamin and trace mineral were supplementation fell below two requirement estimates, but the average of their maximum supplementation was 52.5 and 53.9 times higher than the NRC and China’s Feeding Standard requirements, respectively.

The supplementation rates of vitamins and trace minerals for later-nursery pig diet are reported in Table 3. A total of 64 companies were surveyed, which supplied feed to 502 nursery pig farms. The average fat-soluble vitamin supplementation rate was 2.38 to 12.8 times higher than the NRC estimations, and 2.38 to 15.1 times higher than China’s Feeding Standard. Vitamin D supplementation was 12.8 times higher than the NRC requirement and 15.1 times higher than China’s Feeding Standard. There was substantial variation (SD = 3,103.867) noticed in vitamin A supplementation in this stage. The average added vitamins A, E, and K were close to the requirements. Water-soluble vitamins were supplemented from 0.72 to 7.64 times their NRC requirement estimate, and 0.83 to 7.64 times China’s Feeding Standard. The average supplementation rates of vitamin B_6_, niacin, and choline were lower than NRC values (by 0.82, 0.77, and 0.72 times), although only the amount of choline in diets was lower than China’s Feeding Standard requirement (0.83 times). Trace minerals supplementation was from 1.19 to 20.6 times that of NRC requirement estimate, and from 1.11 to 20.6 times that of China’s Feeding Standard. Iron, zinc, and selenium were supplemented at their required levels, but copper, manganese, and iodine were supplemented well above their requirement estimate (15.4, 20.6, and 11.2 times that of NRC requirement, respectively; 17.1, 20.6, and 11.2 times that of China’s Feeding Standard, respectively). The minimum vitamin and trace mineral supplementation levels were lower than NRC and China’s Feeding Standard estimates, while the average of their maximum supplementation was 113.9 and 124.6 times that of NRC and China’s Feeding Standard, respectively.

### Growing stage

The supplementation rates of vitamins and trace minerals provided by 444 pig farms from 69 producers are presented in Table 4. The average fat-soluble vitamin supplementation rates were from 1.83 to 14.3 times over their NRC requirement estimate, and 1.83 to 13.4 times over China’s Feeding Standard. Among all vitamin categories, vitamin D has the highest ratio to nutrition estimates. The highest variation (SD = 2,295.752) was reported for vitamin A supplementation among these producers. Vitamin E supplementation was on average close to the requirement estimates (1.83 times). Average supplementation rates for vitamins A and K were 3.80 and 5.10 times higher than the NRC estimates, and 3.53 and 5.10 times higher than China’s Feeding Standard. The supplemented water-soluble vitamins basically met growing pigs’ requirements in NRC and China’s Feeding Standard. Water-soluble vitamins were supplemented from 0.56 to 2.48 times that of their NRC requirement estimates, and 0.88 to 2.48 times that of China’s Feeding Standard. The average supplementation rate of niacin was lower than the NRC estimate (0.56 times), and the average supplemented choline was lower than NRC (0.88 times) and China’s Feeding Standard (0.88 times). Trace minerals were supplemented from 1.02 to 28.5 times NRC estimate and China’s Feeding Standard. Zinc and selenium were supplemented according to requirements, and the added copper and iron were close to the requirements of the NRC and China’s Feeding Standard. Manganese and iodine were supplemented well over the requirements, at 28.5 and 11.6 times that of NRC or China’s Feeding Standard, respectively. The minimum vitamin and trace mineral levels were below the requirements for two estimates, while the average of their maximum supplementation was 25.1 and 25.9 times that of NRC and China’s Feeding Standard, respectively.

### Early-finishing stage

The vitamin and trace mineral supplementation rates in early-finishing diet (60 to 90 kg) are shown in Table 5. There were 63 producers surveyed for this feeding stage. These producers supplied early-finishing feed to 417 pig farms. The average fat-soluble vitamin supplementation rate was 2.09 to 13.7 times that of the NRC requirement estimate and China’s Feeding Standard. Vitamin D was supplemented at 13.7 times over the NRC requirement estimate and China’s Feeding Standard. The highest variation (SD = 1,775.468) of vitamin A supplementation was observed at this stage. The average supplementation of vitamins A and E were close to the requirements (3.58 and 2.09 times that of NRC or China’s Feeding Standard, respectively). Water-soluble vitamins were supplemented from 0.50 to 9.54 times their NRC requirement estimate, and 0.63 to 7.95 times China’s Feeding Standard. The average supplementation rate of niacin was lower than NRC estimate, and the average supplemented concentration of choline was lower than NRC and China’s Feeding Standard requirements. Trace minerals supplementation was from 1.24 to 29.1 times higher than the NRC requirement estimate and China’s Feeding Standard requirements. Iron was added at levels 4.56 times higher than the NRC and China’s Feeding Standard requirements. Zinc and selenium were supplemented on average at their requirement estimate, while copper was supplemented at levels 5.26 times greater than their requirement estimation. Manganese and iodine were supplemented well above their requirement estimate, at 29.1 and 11.5 times that of the NRC requirement estimate and China’s Feeding Standard. The minimum values of vitamins A and D were supplemented at NRC requirement estimate and China’s Feeding Standard, but the minimum value of the other vitamins or trace minerals were supplemented below the two estimations. However, the average of their maximum supplementation was 116.9 and 109.3 times higher than the NRC and China’s Feeding Standard requirements, respectively.

### Later-finishing stage

The inclusion rates of vitamins and trace minerals in the later-finishing diet (90 kg to market body weight) are reported in Table 6. These findings were provided by 435 pig farms from 61 producers. The fat-soluble vitamin supplementation rate was 2.17 to 13.6 times that of their NRC requirement estimates and China’s Feeding Standard, respectively. Vitamin D supplementation was 13.4 times that of the NRC requirement estimate and China’s Feeding Standard. Vitamin K supplementation was 13.6 times over the NRC requirement estimate and China’s Feeding Standard. A high amount of variation (SD = 1,772.387) was observed in vitamin A supplementation among producers. Vitamins A and E were supplemented on average at their requirement estimates. Most water-soluble vitamins were fundamentally supplemented to meet finishing pig’s requirements according to the NRC and China’s Feeding Standard, except for niacin and choline. Niacin supplementation was 0.51 times that of the NRC requirement estimate, and choline supplementation was 0.66 times that of the NRC and China’s Feeding Standard requirements. Trace minerals were supplemented at levels from 1.25 to 28.6 times the NRC requirement estimate and China’s Feeding Standard. Zinc and selenium were supplemented to meet the requirement estimates, while copper and iron supplementations were approximately five times over the requirements. Manganese and iodine were supplemented well above their requirement estimate, at 28.6 and 10.6 times that of the NRC and China’s Feeding Standard, respectively. The minimum supplementation of vitamins A and D were at NRC requirement estimate and China’s Feeding Standard levels, and the average of their maximum supplementation was 101.5 and 95.6 times that of NRC and China’s Feeding Standard, respectively.

### Gilt-development stage

The added vitamin and trace mineral concentrations in gilt-development diets were described by 157 farms which are affiliated with 43 producers (Table 7). The average fat-soluble vitamin supplementation rates were 4.14 to 16.7 times that of their NRC requirement estimate, and 4.14 to 14.8 times that of China’s Feeding Standard. Vitamin D was supplemented at 16.7 times that of the NRC requirement estimate and 14.8 times that of China’s Feeding Standard. The supplementation of vitamin A varied (SD = 2,952.290) from producer to producer. The average supplementation rates of vitamins A and E were at the requirement estimate. Most water-soluble vitamins were numerically similar with a pig’s requirements according to the NRC and China’s Feeding Standard, except for niacin, folic acid, and biotin. The added niacin in gilt-development diets was 0.75 times that of the NRC requirement estimate, and folic acid and biotin were supplemented at 7.80 and 11.6 times of their estimated values in NRC and China’s Feeding Standard, respectively. Trace minerals were supplemented from 1.15 to 32.4 times that of the NRC requirement estimate, and 0.99 to 21.6 times of China’s Feeding Standard. Copper, iron, zinc, and selenium were supplemented at the required concentration, and manganese and iodine were supplemented above the required concentration, at 32.4 and 10.3 times that of the NRC guidance and at 21.6 and 10.3 times that of China’s Feeding Standard, respectively. The minimum supplementation of vitamins A and D were at the NRC requirement concentration, while other vitamins and trace minerals were supplemented well below the two estimations of their requirements. The average of their maximum supplementation was 35.2 and 32.7 times that of NRC and China’s Feeding Standard, respectively.

### Gestation, lactation, and boar

The supplementation rates of vitamin and trace minerals in gestating sow diets are displayed in Table 8. The data were provided by 391 farms which were affiliated with 60 producers. The average fat-soluble vitamin supplementation rates were 1.04 to 8.86 times that of their NRC requirement estimates, and 1.14 to 13.6 times that of China’s Feeding Standard. Vitamin K_3_ was supplemented at 8.83 times that of the two requirement estimates. Vitamin D_3_ was supplemented at 13.6 times that of China’s Feeding Standard. The variation of supplemental vitamin A was higher than other ingredients (SD = 2,119.621). Water-soluble vitamins were supplemented from 0.32 to 3.57 times their NRC requirement estimate, and 0.34 to 3.83 times China’s Feeding Standard. The added contents of water-soluble vitamins were similar with the two requirement estimates, but choline was supplemented at 0.32 and 0.34 times of the NRC and China’s Feeding Standard requirements, respectively. Trace minerals were supplemented from 0.72 to 9.40 times that of the NRC requirement estimate, and 1.61 to 10.1 times that of China’s Feeding Standard. Iodine was supplemented at levels well above the requirement estimates, at 9.40 times that of the NRC guidance and 10.1 times that of China’s Feeding Standard. The minimum supplementation values of vitamins and trace minerals were well below the two requirement estimates. The average of their maximum supplementation was 66.5 and 73.3 times that of NRC and China’s Feeding Standard, respectively.

The added vitamin and trace minerals in the lactation diet are shown in Table 9. This information was provided by 387 pig farms from 59 producers. The average fat-soluble vitamin supplementation rates were 1.05 to 8.95 times that of their NRC requirement estimates, and 1.03 to 11.9 times that of China’s Feeding Standard. Vitamin K was supplemented at 8.95 times that of both requirement estimations. Vitamin D was supplemented at 11.9 times that of China’s Feeding Standard. The highest variation was observed within vitamin A supplementation (SD = 1,693.699). Water-soluble vitamins were supplemented from 0.35 to 5.29 times their NRC requirement estimates and China’s Feeding Standard. The average supplementation rate of choline was at 0.35 times that of NRC requirement and China’s Feeding Standard, respectively. Trace minerals were supplemented from 0.72 to 10.3 times that of the NRC requirement estimate, and 1.40 to 10.3 times that of China’s Feeding Standard. Copper, iron, manganese, zinc, and selenium were supplemented at their required levels, while iodine was supplemented 10.3 times over the requirements outlined by the NRC and China’s Feeding Standard. The minimum supplementation values of vitamins and trace minerals were well below requirements. The average of their maximum supplementation was 46.2 and 50.8 times that of NRC and China’s Feeding Standard, respectively.

The supplementation rates of vitamins and trace minerals in boar diets are presented in Table 10. Data were obtained from 101 pig farms affiliated with 40 producers. The average fat-soluble vitamin supplementation rate was 1.13 to 12.5 times that of the NRC requirement estimates, and 1.11 to 11.4 times that of China’s Feeding Standard. Vitamin D was supplemented at level of 12.5 times higher than the NRC requirement estimate, and 11.4 times higher than China’s Feeding Standard. There was a high level of variation (SD = 2,570.465) in vitamin A supplementation among producers. The average inclusion rates of vitamins A and E met the requirements. Most water-soluble vitamins were supplemented to meet requirement estimates of the NRC or China’s Feeding Standard, but choline was supplemented at 0.32 times that of the two feeding standards. Trace minerals were supplemented from 1.30 to 8.78 times that of the NRC requirement estimate, and 0.91 to 8.19 times that of China’s Feeding Standard. Copper, iron, manganese, zinc, and selenium were supplemented at their required levels, and iodine was supplemented above requirements, at 8.78 times that of NRC guidance and 8.19 times that of China’s Feeding Standard, respectively. The minimum vitamin and trace mineral supplementation levels were well below required levels. The average of their maximum supplementation level was 8.66 and 8.59 times over the NRC and China’s Feeding Standard requirements, respectively.

## DISCUSSION

In the present study, a wide range of vitamin and mineral premixes were available to pork producers in China. However, some producers reported excessive dietary vitamin A, vitamin D, and trace minerals supplementation, which was expected. We revealed that producers violated the legal restrictions for vitamins and trace minerals supplementation, which was not the original intention of this study. Further, this may affect the profits of companies and is not key in this discussion section. In terms of the impact of the pig industry on the environment and strict environmental regulations, Chinese nutritionists and producers are focusing on efficiently reducing the excretion of nutrients while maintaining productivity.

We found a high inclusion rate and variation of vitamin A. Vitamin A is extremely important for protection of the epithelium, ovulation, implantation, embryonic and fetal development, immunocompetence, and cell growth and differentiation [14]. The active metabolite of vitamin A, retinoic acid, has a role in a broad spectrum of biological functions, and vitamin A deficiency can severely affect health; thus, commercial sources of vitamin A supplementation are usually kept at a high level. The review by Flohr et al [15] carried out using data obtained from main producers in the United States, found high inclusion rates and variation in vitamin A values. Median vitamin A inclusion rate for piglets and growing–finishing pigs in China is similar to the U.S. [15], but it is lower (by about 0.5 times) than American industry in the breeding herd. Here, the SD of vitamin D ranged widely from 841.848 to 1,726.648, which indicates a very wide range of variation. Our results indicate that in China there are probably two well-defined positions: While some producers use relatively high concentrations of vitamin D, others use a much lower level. The situation in the U.S. is the same: Based on a survey by Flohr et al [15], median vitamin D supplementation in most stages was similar to Chinese producers, but median vitamin D supplementation for growing–finishing stages in China was 2 to 3 times that of the American industry. The main functions of vitamin D include the regulation of calcium and phosphorus absorption and boosting the immune response [4, 16]. The role of vitamin D as an immune system regulator was suggested in early studies with the discovery of expression of vitamin D receptor signaling in immune cells. A previous study reported the early interferon response to rotavirus infection and the effect of vitamin D on host–pathogen interactions. They found that vitamin D supplementation could alleviate the negative effects caused by porcine rotavirus challenge [17]. They observed that supplementation with high concentrations of vitamin D in the diet improved the fecal consistency of the challenged pigs and decreased the secretion of proinflammatory cytokines. This led to high concentrations of vitamin D supplementation in diets. Most terrestrial animals acquire vitamin D by its production in skin under the influence of UV light from the sun. Pigs may not have requirements of vitamin D in dietary sources if sufficient sunlight exposure is provided in modern pig farms [18]; this may be a reason for companies to use low levels of vitamin D in pig diets. In the present study, median vitamin E supplementation in China’s companies was lower than American producers which was reported by Flohr et al [15]. Due to the limited the “space” of formulation for premix, vitamin E supplementation has considerable variation. In addition, vitamin E is a well-known antioxidant and free-radical scavenger [7,18]. The high inclusion rate of vitamin E adopted by some producers was intended to alleviate the stress responses of pigs and improve immune function. A higher level of vitamin E is particularly important for improving lipid stability in meat. However, supplementation of vitamin E should be adjusted according to the unsaturated fatty acid and antioxidant content of feed, and according to the processing and storage of pork. The NRC has established requirements of 0.5 mg vitamin K per kg feed for all ages and productive phases [7]. Limited data have been published on optimal vitamin K supplementation for pigs in good physiological condition and maintained in adequate production conditions. The present survey revealed that vitamin K is commonly and largely added because of the common occurrence of mold toxins in cereal grains, a factor that compromises the blood-clotting mechanism and reduces intestinal synthesis in pigs. In addition, vitamin K is fortified above NRC recommendation, which takes account of degradation of vitamin K during storage and feed processing [12,13]. The variation of this vitamin is relatively high. The situation in the U.S. is different: Flohr et al [15] reported that manufacturers always add high levels of vitamin K in feed, at about 4 to 8 times that of the required levels.

Water-soluble vitamins are not stored in the body and need to be provided continuously to maximize metabolic efficiency and prevent vitamin deficiencies [7,18]. There were low variations in the supplementation rates of thiamine, riboflavin, vitamin B_6_, folic acid, and biotin throughout the life of pigs. Most producers provide these vitamins close to the requirements, and they observed optimum growth under commercial feeding conditions. Previous studies reported that high levels of dietary supplementation of B vitamins could not improve performance, and NRC levels for pigs are sufficient to meet their needs [19,20]. A similar situation was described in a survey carried out in the United States [15], where some producers do not include thiamine, vitamin B_6_, folic acid, and choline (mainly for growing–finishing pigs). The median value in this case for piglets and breeders was 3.1 and 2.2 mg/kg thiamine of feed, respectively; 4.0 and 3.3 mg/kg vitamin B_6_ of feed, respectively; 1.5 and 1.7 mg/kg folic acid of feed, respectively; and 166.8 to 187.0 and 519.2 to 584.1 mg/kg choline of feed, respectively. There is an interesting difference between the situation described in the United States and China. Further, Flohr et al [15] found riboflavin was included in all cases in the United States, and the median value was 1.18 to 1.93 times that of China’s industry. It was observed that the supplementation levels of niacin, vitamin B_6_, and choline in the diets of most life stages were lower than the NRC requirements, but were closed to China’s Feeding Standard requirement. This observation means China’s pigs require lower levels of niacin, vitamin B_6_, and choline from the vitamin premix than American pigs. In fact, Chinese pork producers routinely supplement plant sources or plant by-products in feed, and the abundance of vitamins in these ingredients have been ignored (although some of them have low bioavailability), when vitamin premixes are used as the vitamin source in diets of pigs. In addition, these vitamins have gained little attention from producers, because reducing the level of these vitamins does not affect animal’s performance. Considering the cost of formulation, a low concentration of vitamins is provided to China’s pigs. In addition, the median supplementation rate of vitamin B_12_ in the current case was slightly lower than in the United States, as reported by Flohr et al [15]. The high inclusion rates of vitamin B_12_ reported by some producers were to promote reproduction [21]. This led to a large variation in vitamin B_12_ supplementation rates in the breed phases. Except in the gilt-development period, choline was supplemented below the required level. As a common B vitamin in feed, choline serves as a methyl group donor and a precursor of phosphatidylcholine, which is involved in very low-density lipoprotein assembly in the liver [7,18]. Although the improvement in growth performance and the decrease in liver lipid content as a result of choline supplementation in poultry rations are well established, studies of choline in pigs are limited and the results are inconsistent [18,22]. Thus, there was a high level of variation in choline supplementation rates in each life phase. It is considered that microbial synthesis in the digestive system is sufficient to meet the needs of these animals [1,18]. Nevertheless, when animals are produced in confined conditions, with little possibility of contact with feces, and particularly when animals are young and digestive flora are not well established, administration independent of the animal’s own production is recommended.

Unlike other nutrients, trace elements cannot be generated in the body by *de novo* synthesis [11,23]. Pigs need to regularly ingest them from their diet, or they may deplete their body store and develop deficiency. The high inclusion rates for copper and zinc reported for some producers were used to promote growth and to help control gut pathogens [24,25]. This led to a large variation in copper and zinc supplementation rates. There are environmental concerns associated with such high dietary levels of metals; indeed, some producers adjusted inclusion rates of copper and zinc in their formulation. Flohr et al [15] found that the high inclusion rates of copper and zinc from American producers were used for growth promotion, and the situation in the United States is similar to that of China. The survey by Flohr et al [15] shows that the concentration of iron provided in feed in the United States; the median value of iron in this case is identical to that in China. In this survey, we noticed a high supplementation of manganese from some producers. We speculated that they may be used to improve pork color and reduce oxidant stress. Manganese is a constituent of superoxide dismutase, essential for the synthesis of chondroitin sulfate, and a constituent of mucopolysaccharides in the bone matrix [4,7]. Manganese is a species mineral problem (i.e., poultry) and is therefore of only minor interest to pig health [18], but a study indicated that feeding a supplement of manganese (350 mg/kg of feed) above the maintenance requirements of pigs does not beneficially affect their performance but may improve pork color and delay discoloration of pork during retail display [26]. In a recent article that studied the actual situation of vitamins and trace minerals in American industry, the supplementation rate of manganese was found to be similar in the United States and China. The emergence in the last 10 years of the use of supplemental vitamin E and selenium in pig diets and their roles in the protection of cellular and subcellular membranes from peroxidative damage have been of the utmost importance [7,18]. This may be the most likely reason for the high inclusion rates of selenium in the pig diet. In this study, the 0.3 to 0.4 mg/kg of selenium were supplemented for most pig life stages, which is the same situation as that reported by Flohr et al [15], who found approximately 0.3 mg/kg selenium included in most cases in the United States. This dosage meets the requirement for young pigs, but the selenium requirement for finishing pigs and breeding pigs is about twice as high. In China, there is a legal allowance of 0.5 mg/kg selenium to be included in diet, and this restricts flexibility in terms of addressing the selenium requirements of finishing and breeding pig. Chinese producers routinely add 0.3 to 0.4 mg/kg of selenium in the finishing stage for improving meat quality characteristics such as reducing drip loss and increasing antioxidant stability. Besides, it seems likely that reproducing sows and boars are sensitive to selenium, and selenium plays an important role in the maintenance of semen quality and improved antioxidant defense of sows via specific antioxidant protective mechanisms. In pig production, it is difficult for animals to lack iodine, and low variation of iodine supplementation was observed in the present study. All companies added amounts are below the baseline of legal regulation in pig industry of China. The median inclusion rate of iodine in China’s companies is slightly higher than American companies. Trace minerals are normally supplemented into animal diets as inorganic salts, primarily as oxides, sulfates, and carbonates [11,23]. Although information about the source of these nutrients is not available in the present study, the bioavailability of any given mineral in these salts is generally high, but trace minerals vary with the form of salt. This may be the cause of the variation of trace mineral supplementation among producers.

There has been interest in reducing the supplementation of vitamins and trace minerals from the pig diets. In the current study, the withdrawal of one or more vitamins and trace minerals in diets has been reported. The reason for this is the increasing awareness of the detrimental effects of the excessive minerals to the environment, as well as the soaring cost of vitamins. It has been demonstrated that removing vitamins and trace minerals from some life stages does not affect the pig performance response [27–30]. These are some reasons the lack of performance response resulting from vitamin fortification. The feed ingredients may contain adequate bioavailable levels of one or more vitamins or their precursors [7]; the microorganisms in the digestive tract of adult pigs may synthesize adequate amounts under feeding conditions; dietary prebiotics may alter the intestinal microbiota and their synthesis of vitamins [31]; and the vitamin may be synthesized in sufficient quantities by body tissues in some cases. We believe that the amounts of these vitamins that are present in grains are sufficient for normal growth, but they should be supplemented in diets for breeding pigs. Besides, the average supplementation of vitamins and trace minerals by most producers are relatively close to the commercial vitamin and trace mineral recommendations. Pig producers may wish to include higher levels of vitamin and trace mineral than those listed by the NRC estimates and Feeding Standard of Swine to ensure adequate intake of them and for insurance purposes. In practice, a margin of safety may be added to the stated requirements to account for variability in vitamin and trace mineral content and bioavailability, the presence of inhibitors in ingredients, inadequate processing or mixing of diets, partial loss of vitamins from storage, the impact of environmental stressors on vitamin and trace mineral requirements, and other factors.

## CONCLUSION

The levels of vitamins and trace minerals used or recommended in China’s pig industry by private agencies vary widely within this investigation. Most producers are supplementing at levels relatively close to commercial vitamin and trace mineral recommendations. This survey suggests a baseline of standard practice for trace mineral supplementation rates in different feeding stages in China. With this information, future research still needs to examine the vitamin and trace mineral requirements for various phases of production under large group commercial feeding conditions. In addition, this study may help producers and nutritionists to determine the vitamin and trace mineral requirements which optimize productivity of pig production and maximize economic return.

## Figures and Tables

**Table 1:** The composition of supplemented vitamins and trace minerals in creep diets (weanling to 8 kg)

Item^1)^	N	Average	Ratio to NRC^2)^	Ratio to CHN^3)^	SD	Minimum	The 25th percentile	Median	The 75th percentile	Maximum
Vitamins (unit/kg)										
Vitamin A (IU)	151	10,280	4.67	4.67	3,848.980	1,300	8,080	10,000	12,000	31,850
Vitamin D (IU)	144	3,540	16.1	16.1	1,726.678	150	2,500	3,000	5,150	10,000
Vitamin E (IU)	150	29.4	1.84	1.84	28.461	0.40	11.0	20.0	40.0	210
Vitamin K (mg)	113	4.47	8.95	8.95	9.626	0.03	1.60	2.80	5.27	84.0
Thiamine (mg)	91	2.77	1.85	1.85	4.986	0.04	1.20	2.00	3.00	48.0
Riboflavin (mg)	109	5.95	1.49	1.49	3.598	0.04	4.00	6.00	7.50	32.0
Vitamin B_6_ (mg)	90	3.31	0.47	1.66	2.604	0.30	1.50	2.49	4.50	15.0
Vitamin B_12_ (μg)	70	185	9.25	9.25	884.846	5.00	24.0	32.0	42.0	6,000
Pantothenic acid (mg)	102	21.3	1.77	1.77	14.036	0.24	11.9	16.0	28.5	100.0
Niacin (mg)	89	27.1	0.90	1.35	14.303	1.80	19.8	25.2	35.0	120
Folic acid (mg)	71	2.31	7.70	7.70	5.873	0.20	0.64	1.20	1.63	40.0
Biotin (mg)	64	0.20	2.55	2.55	0.123	0.04	0.11	0.18	0.25	0.80
Choline (mg)	25	448	0.75	0.75	379.264	100	295	300	500	2,000
Trace minerals (mg/kg)										
Copper	138	82.5	13.7	13.7	36.123	3.00	68.4	77.4	103	313
Iron	141	258	2.58	2.45	212.296	19.0	132	184	385	1,270
Manganese	140	70.3	17.6	17.6	30.193	2.00	45.8	76.3	87.4	200
Zinc	141	425	4.25	3.86	498.840	22.8	69.0	100.0	820	1,980
Selenium	79	0.41	1.35	1.35	0.351	0.10	0.30	0.35	0.40	3.00
Iodine	74	1.92	13.7	13.7	1.936	0.10	0.40	0.90	3.62	5.30

The N represents the number of farms; SD, standard deviation.

1) All reported values are on an as-fed basis and represent vitamins and trace minerals provided the following per kilogram of diet for pigs.

2) Values represent average supplementation rates as a proportion to dietary vitamin and trace mineral requirements recommendation from the NRC [7].

3) Values represent average supplementation rates as a proportion to dietary vitamin and trace mineral requirements recommendation from the China’s Feeding Standard of Swine [9].

**Table 2:** The composition of supplemented vitamins and trace minerals in early nursery diets (8 to 15 kg)

Item^1)^	N	Average	Ratio to NRC^2)^	Ratio to CHN^3)^	SD	Minimum	The 25th percentile	Median	The 75th percentile	Maximum
Vitamins (unit/kg)										
Vitamin A (IU)	312	10,186	4.63	5.66	3,124.882	700	8,750	10,000	11,750	31,850
Vitamin D (IU)	303	2,828	12.9	14.1	1,239.169	150	2,200	2,900	3,200	10,000
Vitamin E (IU)	310	33.9	2.12	3.08	31.953	0.40	15.2	22.0	41.0	240
Vitamin K (mg)	263	3.46	6.91	6.91	6.402	0.03	2.00	2.75	3.75	84.0
Thiamine (mg)	194	2.67	2.67	2.67	4.803	0.04	1.20	2.00	3.19	48.0
Riboflavin (mg)	273	5.80	1.66	1.66	3.777	0.04	4.00	4.80	7.20	40.0
Vitamin B_6_ (mg)	224	2.91	0.42	1.94	2.317	0.02	1.50	2.30	4.00	20.0
Vitamin B_12_ (μg)	140	106	6.08	6.08	628.468	1.50	18.3	29.9	38.4	6,000
Pantothenic acid (mg)	236	19.3	1.93	1.93	11.839	0.18	10.5	16.0	24.9	100.0
Niacin (mg)	218	26.4	0.88	1.76	14.766	0.20	17.2	25.0	36.0	120
Folic acid (mg)	123	2.38	7.95	7.95	6.099	0.03	0.58	0.90	1.80	40.0
Biotin (mg)	130	0.19	3.73	3.73	0.106	0.01	0.11	0.18	0.25	0.80
Choline (mg)	70	343	0.69	0.69	244.121	60.0	240	300	399	2,000
Trace minerals (mg/kg)										
Copper	278	76.6	12.8	12.8	31.900	3.00	64.7	77.5	92.5	313
Iron	290	244	2.44	2.32	155.763	0.13	130	200	371	1,270
Manganese	285	62.6	15.7	15.7	27.680	0.03	40.0	64.0	80.0	200
Zinc	286	534	5.34	4.86	494.257	0.12	80.0	133.3	913	1,980
Selenium	164	0.37	1.23	1.23	0.315	0.09	0.24	0.32	0.39	3.00
Iodine	159	1.25	8.91	8.91	1.410	0.10	0.40	0.60	1.28	5.20

The N represents the number of farms; SD, standard deviation.

1) All reported values are on an as-fed basis and represent vitamins and trace minerals provided the following per kilogram of diet for pigs.

2) Values represent average supplementation rates as a proportion to dietary vitamin and trace mineral requirements recommendation from the NRC [7].

3) Values represent average supplementation rates as a proportion to dietary vitamin and trace mineral requirements recommendation from the China’s Feeding Standard of Swine [9].

**Table 3:** The composition of supplemented vitamins and trace minerals in late nursery diets (15 to 25 kg)

Item^1)^	N	Average	Ratio to NRC^2)^	Ratio to CHN^3)^	SD	Minimum	The 25th percentile	Median	The 75th percentile	Maximum
Vitamins (unit/kg)										
Vitamin A (IU)	488	9,690	5.54	6.46	3,103.867	350	8,500	9,600	11,000	31,850
Vitamin D (IU)	478	2,565	12.8	15.1	1,227.258	150	1,980	2,600	3,000	12,740
Vitamin E (IU)	482	26.2	2.38	2.38	22.286	0.04	14.0	21.0	30.0	240
Vitamin K (mg)	432	3.33	6.67	6.67	5.096	0.03	2.00	2.50	3.98	84.0
Thiamine (mg)	286	2.02	2.02	2.02	2.976	0.01	1.00	1.60	2.40	48.0
Riboflavin (mg)	427	5.01	1.67	2.00	3.243	0.04	3.50	4.20	6.00	40.0
Vitamin B_6_ (mg)	365	2.47	0.82	2.47	2.334	0.02	1.20	1.80	2.94	19.2
Vitamin B_12_ (μg)	226	69.7	4.65	6.34	495.872	0.40	14.0	20.0	28.9	6,000
Pantothenic acid (mg)	387	16.3	1.81	2.04	10.401	0.13	10.5	12.8	20.0	100.0
Niacin (mg)	347	23.2	0.77	2.32	13.831	0.20	16.0	20.0	30.0	120
Folic acid (mg)	207	1.33	4.42	4.42	3.641	0.06	0.40	0.65	1.25	40.0
Biotin (mg)	213	0.38	7.64	7.64	3.280	0.01	0.08	0.15	0.20	48.0
Choline (mg)	139	289	0.72	0.83	204.247	0.28	200	240	320	2,000
Trace minerals (mg/kg)										
Copper	451	76.8	15.4	17.1	51.610	0.13	63.8	76.0	88.5	649
Iron	460	240	2.40	3.43	138.499	0.12	114	210	394	660
Manganese	458	61.9	20.6	20.6	27.100	0.03	40.0	64.0	81.2	250
Zinc	455	95.2	1.19	1.36	143.662	0.12	64.5	76.5	84.2	1,980
Selenium	295	0.33	1.34	1.11	0.244	0.10	0.24	0.30	0.35	3.00
Iodine	290	1.57	11.2	11.2	1.706	0.09	0.40	0.74	2.03	7.66

The N represents the number of farms; SD, standard deviation.

1) All reported values are on an as-fed basis and represent vitamins and trace minerals provided the following per kilogram of diet for pigs.

2) Values represent average supplementation rates as a proportion to dietary vitamin and trace mineral requirements recommendation from the NRC [7].

3) Values represent average supplementation rates as a proportion to dietary vitamin and trace mineral requirements recommendation from the China’s Feeding Standard of Swine [9].

**Table 4:** The composition of supplemented vitamins and trace minerals in growing diets (25 to 60 kg)

Item^1)^	N	Average	Ratio to NRC^2)^	Ratio to CHN^3)^	SD	Minimum	The 25th percentile	Median	The 75th percentile	Maximum
Vitamins (unit/kg)										
Vitamin A (IU)	428	4,946	3.80	3.53	2,295.752	1,300	4,050	4,695	5,250	31,000
Vitamin D (IU)	424	2,145	14.3	13.4	1,071.845	0.29	1,480	2,400	2,750	11,600
Vitamin E (IU)	427	20.1	1.83	1.83	24.089	0.40	10.4	15.6	20.8	240
Vitamin K (mg)	379	2.55	5.10	5.10	1.833	0.03	1.20	1.65	4.10	8.80
Thiamine (mg)	233	1.20	1.20	1.20	0.681	0.004	0.87	1.04	1.58	6.60
Riboflavin (mg)	378	3.42	1.37	1.71	1.654	0.04	2.40	3.00	4.02	19.8
Vitamin B_6_ (mg)	297	1.55	1.55	1.55	1.542	0.02	0.78	1.00	2.00	16.0
Vitamin B_12_ (μg)	185	14.8	1.48	1.85	8.394	2.40	10.0	15.0	19.2	96.0
Pantothenic acid (mg)	342	12.4	1.54	1.65	8.119	0.10	7.4	10.0	16.0	97.0
Niacin (mg)	299	16.7	0.56	1.97	12.697	0.06	10.0	17.0	20.0	200
Folic acid (mg)	159	0.74	2.48	2.48	1.858	0.08	0.30	0.40	0.64	20.0
Biotin (mg)	146	0.12	2.33	2.33	0.077	0.01	0.07	0.09	0.15	0.48
Choline (mg)	119	263	0.88	0.88	235.787	20.0	160	200	300	2,000
Trace minerals (mg/kg)										
Copper	422	21.1	5.28	5.28	24.748	3.00	14.0	15.5	17.5	200
Iron	432	230	3.83	3.83	134.905	0.13	111	184	380	638
Manganese	425	57.0	28.5	28.5	24.278	0.03	35.0	60.0	77.5	137
Zinc	423	61.0	1.02	1.02	26.212	0.12	50.0	58.4	63.6	275
Selenium	282	0.32	1.59	1.27	0.181	0.10	0.24	0.30	0.33	1.30
Iodine	278	1.63	11.6	11.6	1.794	0.10	0.38	0.78	2.10	7.65

The N represents the number of farms; SD, standard deviation.

1) All reported values are on an as-fed basis and represent vitamins and trace minerals provided the following per kilogram of diet for pigs.

2) Values represent average supplementation rates as a proportion to dietary vitamin and trace mineral requirements recommendation from the NRC [7].

3) Values represent average supplementation rates as a proportion to dietary vitamin and trace mineral requirements recommendation from the China’s Feeding Standard of Swine [9].

**Table 5:** The composition of supplemented vitamins and trace minerals in early-finishing diets (60 to 90 kg)

Item^1)^	N	Average	Ratio to NRC^2)^	Ratio to CHN^3)^	SD	Minimum	The 25th percentile	Median	The 75th percentile	Maximum
Vitamins (unit/kg)										
Vitamin A (IU)	405	4,656	3.58	3.58	1,775.468	1,300	3,930	4,600	5,073	22,400
Vitamin D (IU)	404	2,058	13.7	13.7	963.415	150	1,320	2,400	2,740	6,370
Vitamin E (IU)	402	23.0	2.09	2.09	30.138	0.80	10.0	12.6	22.1	240
Vitamin K (mg)	355	4.94	9.87	9.87	29.978	0.20	1.05	1.60	4.16	400
Thiamine (mg)	227	1.17	1.17	1.17	0.707	0.004	0.70	1.00	1.60	7.50
Riboflavin (mg)	358	3.16	1.58	1.58	1.414	0.30	2.20	2.56	4.00	8.00
Vitamin B_6_ (mg)	277	1.54	1.54	1.54	1.989	0.02	0.64	1.00	1.92	16.0
Vitamin B_12_ (μg)	174	47.7	9.54	7.95	319.338	0.01	8.00	12.0	18.3	3,000
Pantothenic acid (mg)	301	10.7	1.53	1.53	5.972	0.10	5.60	8.55	16.0	36.0
Niacin (mg)	263	15.0	0.50	2.00	7.455	0.48	9.60	15.0	20.0	45.0
Folic acid (mg)	144	0.95	3.16	3.16	2.934	0.08	0.22	0.34	0.60	20.0
Biotin (mg)	137	0.29	5.89	5.89	1.085	0.02	0.06	0.09	0.16	10.0
Choline (mg)	140	188	0.63	0.63	100.018	0.10	120	182	240	480
Trace minerals (mg/kg)										
Copper	393	18.4	5.26	5.26	19.535	1.43	14.0	15.5	17.5	200
Iron	400	228	4.56	4.56	142.305	0.13	99.1	199	389	638
Manganese	395	58.3	29.1	29.1	28.666	0.03	33.0	60.0	79.0	334
Zinc	394	61.8	1.24	1.24	54.888	0.12	48.0	58.8	62.3	1,075
Selenium	274	0.37	2.46	1.47	1.115	0.03	0.24	0.30	0.32	18.6
Iodine	258	1.61	11.5	11.5	1.814	0.03	0.38	0.78	1.77	7.67

The N represents the number of farms; SD, standard deviation.

1) All reported values are on an as-fed basis and represent vitamins and trace minerals provided the following per kilogram of diet for pigs.

2) Values represent average supplementation rates as a proportion to dietary vitamin and trace mineral requirements recommendation from the NRC [7].

3) Values represent average supplementation rates as a proportion to dietary vitamin and trace mineral requirements recommendation from the China’s Feeding Standard of Swine [9].

**Table 6:** The composition of supplemented vitamins and trace minerals in late finishing diets (90 kg to market)

Item^1)^	N	Average	Ratio to NRC^2)^	Ratio to CHN^3)^	SD	Minimum	The 25th percentile	Median	The 75th percentile	Maximum
Vitamins (unit/kg)										
Vitamin A (IU)	420	4,826	3.71	3.71	1,772.387	1,300	4,000	4,700	5,250	22,400
Vitamin D (IU)	423	2,005	13.4	13.4	914.246	150	1,300	2,160	2,690	6,000
Vitamin E (IU)	419	23.9	2.17	2.17	31.285	0.80	10.0	12.8	24.0	205
Vitamin K (mg)	375	6.79	13.6	13.6	41.169	0.20	1.00	1.60	3.80	400
Thiamine (mg)	242	1.19	1.19	1.19	0.671	0.004	0.76	1.11	1.60	7.50
Riboflavin (mg)	379	3.22	1.61	1.61	1.353	0.30	2.24	2.80	4.00	8.00
Vitamin B_6_ (mg)	290	1.54	1.54	1.54	1.724	0.02	0.64	1.00	2.00	16.0
Vitamin B_12_ (μg)	194	29.1	5.82	4.85	214.499	0.01	9.15	12.2	19.1	3,000
Pantothenic acid (mg)	328	10.8	1.54	1.54	5.931	0.10	5.60	8.63	16.0	36.0
Niacin (mg)	286	15.3	0.51	2.04	7.305	0.48	10.0	15.0	19.6	45.0
Folic acid (mg)	155	0.77	2.58	2.58	2.129	0.08	0.23	0.36	0.65	16.0
Biotin (mg)	167	0.18	3.53	3.53	0.509	0.02	0.06	0.08	0.16	6.40
Choline (mg)	144	197	0.66	0.66	109.868	0.10	120	195	240	520
Trace minerals (mg/kg)										
Copper	386	18.5	6.17	5.29	19.710	1.43	14.0	15.5	17.5	200
Iron	394	229	5.72	4.58	139.995	0.13	101	200	383	638
Manganese	388	57.3	28.6	28.6	25.087	0.03	33.3	60.0	80.0	136
Zinc	388	62.5	1.25	1.25	55.358	0.12	48.9	60.0	63.3	1,075
Selenium	256	0.30	2.02	1.21	0.150	0.03	0.24	0.30	0.33	1.20
Iodine	244	1.48	10.6	10.6	1.723	0.03	0.38	0.75	1.38	7.67

The N represents the number of farms; SD, standard deviation.

1) All reported values are on an as-fed basis and represent vitamins and trace minerals provided the following per kilogram of diet for pigs.

2) Values represent average supplementation rates as a proportion to dietary vitamin and trace mineral requirements recommendation from the NRC [7].

3) Values represent average supplementation rates as a proportion to dietary vitamin and trace mineral requirements recommendation from the China’s Feeding Standard of Swine [9].

**Table 7:** The composition of supplemented vitamins and trace minerals in gilt-development diets

Item^1)^	N	Average	Ratio to NRC^2)^	Ratio to CHN^3)^	SD	Minimum	The 25th percentile	Median	The 75th percentile	Maximum
Vitamins (unit/kg)										
Vitamin A (IU)	151	6,640	5.11	4.43	2,952.290	1,300	4,800	6,650	7,800	27,660
Vitamin D (IU)	151	2,512	16.7	14.8	1,494.768	150	1,900	2,600	3,000	17,750
Vitamin E (IU)	151	45.5	4.14	4.14	38.031	2.50	20.0	42.0	50.0	240
Vitamin K (mg)	131	3.01	6.02	6.02	1.793	0.43	2.00	2.40	4.55	7.60
Thiamine (mg)	66	1.31	1.31	1.31	0.616	0.01	0.98	1.11	1.71	2.60
Riboflavin (mg)	132	4.55	1.82	1.82	1.945	1.80	3.00	4.32	5.49	10.0
Vitamin B_6_ (mg)	115	2.57	2.57	2.57	2.273	0.39	1.20	2.44	3.20	18.0
Vitamin B_12_ (μg)	46	14.7	1.47	1.33	6.856	5.00	8.30	16.0	20.0	31.5
Pantothenic acid (mg)	113	16.1	2.01	2.01	7.820	0.22	10.1	14.5	20.0	45.0
Niacin (mg)	96	22.6	0.75	2.26	10.951	2.00	14.6	22.0	31.5	50.0
Folic acid (mg)	49	2.34	7.80	7.80	4.117	0.06	0.32	0.75	3.75	27.0
Biotin (mg)	45	0.58	11.6	11.6	1.630	0.04	0.09	0.20	0.40	8.00
Choline (mg)	59	349	1.16	1.00	175.161	50.0	180	350	500	680
Trace minerals (mg/kg)										
Copper	156	20.7	5.19	4.61	42.781	3.00	14.0	15.0	17.5	393
Iron	154	236	3.93	3.37	128.045	0.13	116	230	382	465
Manganese	152	64.8	32.4	21.6	24.034	0.04	41.3	69.0	85.0	103
Zinc	152	69.3	1.15	0.99	13.894	30.0	58.4	70.0	78.0	123
Selenium	107	0.36	1.79	1.20	0.263	0.10	0.24	0.30	0.35	1.50
Iodine	115	1.44	10.3	10.3	1.692	0.10	0.40	0.60	1.20	5.18

The N represents the number of farms; SD, standard deviation.

1) All reported values are on an as-fed basis and represent vitamins and trace minerals provided the following per kilogram of diet for pigs.

2) Values represent average supplementation rates as a proportion to dietary vitamin and trace mineral requirements recommendation from the NRC [7].

3) Values represent average supplementation rates as a proportion to dietary vitamin and trace mineral requirements recommendation from the China’s Feeding Standard of Swine [9].

**Table 8:** The composition of supplemented vitamins and trace minerals in gestation diets

Item^1)^	N	Average	Ratio to NRC^2)^	Ratio to CHN^3)^	SD	Minimum	The 25th percentile	Median	The 75th percentile	Maximum
Vitamins (unit/kg)										
Vitamin A (IU)	379	7,721	1.93	2.13	2,119.621	480	6,650	8,000	9,000	22,400
Vitamin D (IU)	380	2,445	3.06	13.6	918.711	150	1,928	2,500	3,086	11,900
Vitamin E (IU)	376	45.7	1.04	1.14	31.474	0.80	20.2	45.0	60.0	240
Vitamin K (mg)	345	4.43	8.86	8.86	22.112	0.45	2.00	2.40	3.64	400
Thiamine (mg)	215	1.49	1.49	1.66	0.669	0.01	1.00	1.50	1.68	4.55
Riboflavin (mg)	343	4.68	1.25	1.38	1.663	0.90	3.60	4.80	5.60	10.0
Vitamin B_6_ (mg)	295	2.44	2.44	2.71	1.528	0.02	1.28	2.72	3.20	16.0
Vitamin B_12_ (μg)	160	53.6	3.57	3.83	315.826	1.60	14.6	20.0	22.4	3,500
Pantothenic acid (mg)	311	17.2	1.43	1.56	7.749	0.18	12.0	16.0	20.0	42.8
Niacin (mg)	286	23.2	2.32	2.56	9.765	0.60	16.0	24.0	28.0	75.0
Folic acid (mg)	161	2.37	1.83	1.98	2.450	0.14	0.75	1.60	4.00	22.1
Biotin (mg)	164	0.43	2.16	2.27	0.791	0.03	0.20	0.32	0.43	8.00
Choline (mg)	129	394	0.32	0.34	342.862	0.40	260	364	480	3,840
Trace minerals (mg/kg)										
Copper	366	18.1	1.81	3.62	29.129	1.43	14.0	15.2	17.5	393
Iron	370	232	2.90	3.09	132.764	0.12	112	225	380	465
Manganese	365	62.7	2.51	3.48	25.114	0.04	40.0	66.0	84.0	113
Zinc	365	72.4	0.72	1.61	29.116	0.12	60.0	70.0	78.0	530
Selenium	235	0.35	2.35	2.52	0.282	0.03	0.24	0.30	0.35	2.50
Iodine	237	1.32	9.40	10.1	1.515	0.03	0.40	0.64	1.20	5.35

The N represents the number of farms; SD, standard deviation.

1) All reported values are on an as-fed basis and represent vitamins and trace minerals provided the following per kilogram of diet for pigs.

2) Values represent average supplementation rates as a proportion to dietary vitamin and trace mineral requirements recommendation from the NRC [7].

3) Values represent average supplementation rates as a proportion to dietary vitamin and trace mineral requirements recommendation from the China’s Feeding Standard of Swine [9].

**Table 9:** The composition of supplemented vitamin and trace minerals in lactation diets

Item^1)^	N	Average	Ratio to NRC^2)^	Ratio to CHN^3)^	SD	Minimum	The 25th percentile	Median	The 75th percentile	Maximum
Vitamins (unit/kg)										
Vitamin A (IU)	374	5,457	2.73	2.66	1,693.699	350	4,700	5,425	5,953	22,400
Vitamin D (IU)	374	2,437	3.05	11.9	904.496	150	1,838	2,575	3,100	6,860
Vitamin E (IU)	372	46.3	1.05	1.03	32.539	0.08	22.8	45.0	60.0	240
Vitamin K (mg)	332	4.47	8.95	8.95	14.920	0.45	2.00	2.58	4.55	200
Thiamine (mg)	208	1.48	1.48	1.48	0.735	0.01	1.00	1.40	1.80	5.00
Riboflavin (mg)	337	4.70	1.25	1.22	1.666	1.44	3.60	4.80	5.78	11.0
Vitamin B_6_ (mg)	287	2.40	2.40	2.40	1.647	0.02	1.20	2.50	3.20	18.0
Vitamin B_12_ (μg)	146	79.4	5.29	5.29	436.557	0.40	12.0	19.6	23.0	3,500
Pantothenic acid (mg)	301	17.0	1.42	1.42	7.366	0.19	12.2	16.0	19.1	40.0
Niacin (mg)	279	23.6	2.36	2.30	11.698	0.60	16.0	24.0	30.0	99.0
Folic acid (mg)	147	2.45	1.89	1.82	2.988	0.14	0.80	1.40	3.40	22.1
Biotin (mg)	148	0.36	1.79	1.70	0.545	0.03	0.20	0.29	0.42	6.40
Choline (mg)	138	350	0.35	0.35	164.536	0.40	240	354	480	700
Trace minerals (mg/kg)										
Copper	356	18.1	0.90	3.62	28.707	1.43	14.0	15.5	17.5	393
Iron	362	235	2.94	2.94	137.445	0.12	116	203	390	790
Manganese	356	63.6	2.54	3.18	25.667	0.03	42.1	69.0	85.0	166
Zinc	355	71.6	0.72	1.40	26.678	0.12	60.0	70.0	78.0	460
Selenium	223	0.35	2.35	2.35	0.285	0.03	0.25	0.30	0.35	2.50
Iodine	226	1.44	10.3	10.3	1.704	0.03	0.40	0.71	1.21	10.2

The N represents the number of farms; SD, standard deviation.

1) All reported values are on an as-fed basis and represent vitamins and trace minerals provided the following per kilogram of diet for pigs.

2) Values represent average supplementation rates as a proportion to dietary vitamin and trace mineral requirements recommendation from the NRC [7].

3) Values represent average supplementation rates as a proportion to dietary vitamin and trace mineral requirements recommendation from the China’s Feeding Standard of Swine [9].

**Table 10:** The composition of supplemented vitamin and trace minerals in boar diets

Item^1)^	N	Average	Ratio to NRC^2)^	Ratio to CHN^3)^	SD	Minimum	The 25th percentile	Median	The 75th percentile	Maximum
Vitamins (unit/kg)										
Vitamin A (IU)	99	7,071	1.77	1.77	2,570.465	1,300	5,500	6,650	8,800	14,000
Vitamin D (IU)	99	2,500	12.5	11.4	841.848	150	2,000	2,600	3,075	4,000
Vitamin E (IU)	99	49.9	1.13	1.11	36.291	3.00	24.0	48.0	60.0	240
Vitamin K (mg)	87	3.08	6.16	6.16	1.800	0.45	2.00	2.50	3.80	10.4
Thiamine (mg)	60	1.45	1.45	1.45	0.624	0.40	1.00	1.42	1.78	3.00
Riboflavin (mg)	90	4.66	1.24	1.33	1.697	1.60	3.85	4.80	6.00	8.00
Vitamin B_6_ (mg)	73	2.25	2.25	2.25	1.178	0.54	1.16	2.34	3.19	5.00
Vitamin B_12_ (μg)	41	17.0	1.13	1.13	6.128	5.00	13.6	20.0	20.0	28.0
Pantothenic acid (mg)	73	19.0	1.59	1.59	8.459	7.20	13.0	16.0	27.2	45.0
Niacin (mg)	77	21.4	2.14	2.14	7.579	3.60	15.6	20.8	28.0	35.0
Folic acid (mg)	41	2.94	2.26	2.26	4.666	0.22	0.64	1.28	5.00	28.8
Biotin (mg)	39	0.30	1.51	1.51	0.182	0.04	0.20	0.26	0.40	0.80
Choline (mg)	30	401	0.32	0.32	130.690	200	280	400	485	700
Trace minerals (mg/kg)										
Copper	99	15.8	3.15	3.15	3.304	3.00	13.8	16.0	17.5	27.0
Iron	99	237	2.96	2.96	143.529	0.13	120	210	387	760
Manganese	98	61.7	3.09	3.09	26.134	0.03	40.0	62.0	80.0	160
Zinc	97	68.5	1.37	0.91	22.688	30.0	57.0	64.0	70.0	220
Selenium	65	0.39	1.30	2.61	0.660	0.10	0.26	0.32	0.37	1.35
Iodine	64	1.23	8.78	8.19	1.479	0.10	0.38	0.56	1.11	5.18

The N represents the number of farms; SD, standard deviation.

1) All reported values are on an as-fed basis and represent vitamins and trace minerals provided the following per kilogram of diet for pigs.

2) Values represent average supplementation rates as a proportion to dietary vitamin and trace mineral requirements recommendation from the NRC [7].

3) Values represent average supplementation rates as a proportion to dietary vitamin and trace mineral requirements recommendation from the China’s Feeding Standard of Swine [9].
